# Carbapenem-Resistant *E. cloacae* in Southwest China: Molecular Analysis of Resistance and Risk Factors for Infections Caused by NDM-1-Producers

**DOI:** 10.3389/fmicb.2018.00658

**Published:** 2018-04-04

**Authors:** Xiaojiong Jia, Wei Dai, Weijia Ma, Jinrong Yan, Jianchun He, Shuang Li, Congya Li, Shuangshuang Yang, Xiuyu Xu, Shan Sun, Jing Shi, Liping Zhang

**Affiliations:** Department of Laboratory Medicine, The First Affiliated Hospital of Chongqing Medical University, Chongqing, China

**Keywords:** carbapenems, resistance, *E. cloacae*, outbreak investigation, risk factor

## Abstract

Carbapenem-resistant *Enterobacteriaceae* (CRE) has been considered a serious global threat, but carbapenem resistance remains relatively uncommon in *E. cloacae*, especially in China. The aim of this study was to characterize carbapenem-resistant *E. cloacae* (CR-ECL) isolates from 2012 to 2016 in Southwest China. Our study revealed that 20 (15.2%) of the 132 CR-ECL isolates obtained from patients were identified as NDM-1, with most isolates carrying the IncFIIA plasmids. Notably, we initially observed that the *E. cloacae* strain co-harbored NDM-1 and IMP-8 carbapenemases simultaneously. Analysis of the genetic environment of these two genes has revealed that the highly conserved regions (*bl*a_NDM-1_-*ble*_MBL_-*trpF*-*tat*) are associated with the dissemination of NDM-1, while IS26, *intI1*, and *tniC* could be involved in the spread of IMP-8. Molecular epidemiology studies showed the nosocomial outbreak caused by NDM-1-producing *E. cloacae* ST88. Transferring from another hospital and previous carbapenem exposure were identified as independent risk factors for the acquisition of NDM-1-producing *E. cloacae.* These findings emphasize the need for intensive surveillance and precautions to monitor the further spread of NDM-1 in China.

## Introduction

**FIGURE 1:**
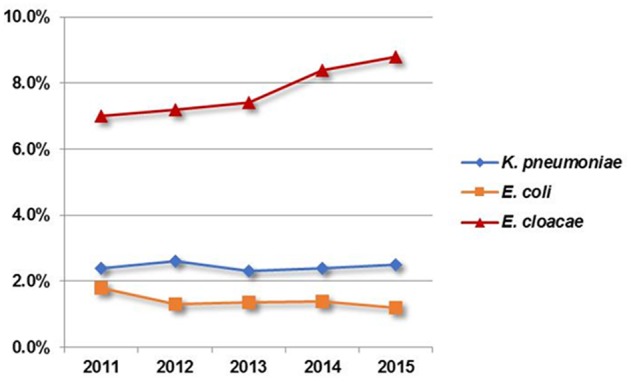
Distribution of carbapenem resistant *Enterobacteriaceae* from 2011 to 2015 according to the Chongqing Resistance Monitoring Network.

As the utility of carbapenem has increased worldwide over the last decade, the emergence and dissemination of carbapenem-resistant *Enterobacteriaceae* (CRE) have become worsening situations (Cornaglia et al., 2011). Recent surveillance data from the United States, Europe, and South Asia has observed a high resistance rate in CRE, among which *Klebsiella pneumoniae* and *Escherichia coli* were the most prevalent (Nordmann et al., 2009; Pollett et al., 2014; Hsu et al., 2017). Similar results were obtained from the CHINET surveillance system, which showed that carbapenem-resistant *K. pneumoniae* (CRKP) appeared to be most common in China and increased rapidly from 9.4% in 2011 to 15.6% in 2015 (Tian et al., 2016). Unexpectedly, however, surveys released by the Chongqing Resistance Monitoring Network (CQRMN) found that the rate of carbapenem resistance showed the highest in *E. cloacae* among CRE isolates during the period 2011–2015, while resistance rates for *K. pneumoniae* and *E. coli* remained relatively stable and had an average of 2.4 and 1.4%, respectively, compared to 7.8% for *E. cloacae* (**Figure 1**). Therefore, investigating carbapenem-resistant *E. cloacae* is of utmost importance for therapy and control in our region.

In China, the first CR-ECL was a KPC-producing strain isolated from Shanghai in 2010 (Wu et al., 2010). Later, carbapenemases IMP, VIM, and, recently, NDM have been reported in clinical *E. cloacae* isolates from different geographical regions (Dai et al., 2013; Yang et al., 2014; Liu et al., 2015), among which NDM-1 should be especially worrisome as the gene encoding this enzyme is often located on mobile genetic elements that can be easily transferred between different species (Rasheed et al., 2013). To date, there have been few studies of outbreak due to NDM-1 producing *E. cloacae* (Stoesser et al., 2015; Mahida et al., 2017); however, to the best of our knowledge, there are no previous studies of nosocomial outbreaks with *E. cloacae* that produce NDM-1 in China. More importantly, strains co-producing various carbapenemases are worth being concerned about, since these bacteria may confer a higher-level resistance to carbapenem that further reduces the therapeutic choices. To date, there has been no report of clinical *E. cloacae* strains simultaneously producing NDM-1 and IMP-8 carbapenemases, and knowledge gaps regarding the genetic context and plasmid characterization of these isolates remain. Additionally, the risk factors and clinical outcomes of nosocomial patients with NDM-1 producing *E. cloacae* have not been systematically evaluated.

Therefore, the objectives of this study were the following: (i) to describe the prevalence of clinical CR-ECL isolates collected successively for approximately 4 years, (ii) to identify the mechanisms and clonal relatedness among these CR-ECL strains, (iii) to report the first outbreak of NDM-1 producing *E. cloacae* in China, (iv) to examine the genetic context of NDM-1 and IMP-8, and (v) to evaluate the risk factors and clinical outcomes of NDM-1 positive *E. cloacae* infections in hospitalized patients.

## Materials and Methods

### Study Setting and Bacterial Strains

This retrospective study was performed in a 3,200-bed tertiary university-affiliated hospital and associated two branch hospitals located in Southwest China. Between March 2012 and 2016, 1,146 clinical *E. cloacae* strains were collected in these hospitals. We chose the first isolate from the patient and excluded duplicate isolates from the same patient. All isolates were identified at the species level and routine antimicrobial susceptibility testing was performed by using the VITEK2 compact or VITEK MS (bioMerieux, Hazelwood, MO, United States) automated system. The isolates were collected by the rapid freezing method and stored at -80°C for further analysis. Isolates were included in the study if they were resistant to at least one of the carbapenems by the broth microdilution method, with the criteria of MICs of ≥ 2 μg/mL for ertapenem, ≥ 4 μg/mL for imipenem, or ≥ 4 μg/mL for meropenem. For research involving biohazards, the standard biosecurity or institutional safety procedures have been carried out.

### Antimicrobial Susceptibility Testing

All isolates were tested for antibiotic susceptibility to ceftazidime (CAZ), ceftriaxone (CRO), cefepime (FEP), gentamicin (GM), tobramycin (TOB), ciprofloxacin (CIP), and levofloxacin (LEV) by using AST GN13 cards on the VITEK2 compact system. MICs of ertapenem (ETP), imipenem (IPM), meropenem (MEM), colistin (CS), and tigecycline (TGC) were determined using the broth microdilution method, and results were interpreted according to the CLSI M100-S25 interpretive criteria [Clinical and Laboratory Standards Institute [CLSI], 2015]. *E. cloacae* ATCC 13847 was used as the quality control strain for susceptibility testing. MIC_50_, MIC_90_, and the MIC range of each tested agent were also analyzed in our study.

### Detection of Antibiotic-Resistant Genes, Expression of Outer Membrane Proteins, and Activity of the Efflux Pump

The PCR was performed to detect for the presence of carbapenemase-related genes, including *bla*_KPC_, *bla*_NDM_, *bla*_V IM_, *bla*_IMP_, *bla*_SME_, and *bla*_OXA-48_, and sequencing was used to confirm the variants of these carbapenemase genes. Moreover, ESBLs, AmpC, resistant genes for aminoglycosides and fluoroquinolones, *ompF* and *ompC* genes were also determined by using primers as described previously (Doumith et al., 2009; Zhang et al., 2014). Additionally, the levels of expression of porin-encoding genes were determined from mRNA levels by real-time reverse transcription (RT)-PCR according to the previously described protocols (Bustin et al., 2009). The activity of the efflux pump was examined by comparing the MICs to ertapenem among resistant isolates in the presence and absence of carbonyl cyanide *m*-chlorophenylhydrazone (CCCP) as an efflux pump inhibitor (Chollet et al., 2004). A twofold decrease in MIC after the addition of CCCP was considered positive results. Plates containing CCCP but no carbapenem were used as controls.

### Conjugation, Transformation, and Plasmid Analysis

To assess whether the carbapenemase-producing genes were located on the plasmids and to assess the transferability of these genes, the co-producing *bla*_NDM-1_ and *bla*_IMP-8_ strains (ECL-86) were conducted by conjugation with *E. coli* EC600 and by transformation with *E. coli* DH5α. For conjugative assays, transconjugants were selected on Mueller-Hinton agar plates supplemented with a combination of 8 mg/L ampicillin and 256 mg/L rifampicin. For transformative assays, transformants were selected on Mueller-Hinton agar plates containing 64 mg/L ampicillin. The transconjugants and transformants were tested for antimicrobial susceptibility by the VITEK2 compact system, and they confirmed the presence of resistance determinants by PCR. In addition, all NDM-1-encoding plasmids were characterized for the incompatibility groups by using the PCR-based replicon typing method described previously (Carattoli et al., 2005).

### Genetic Environments of NDM-1-Carrying Plasmids and IMP-8-Carrying Plasmids

To investigate the genetic contests in the NDM-1 and IMP-8, we selected 14 representative strains producing single NDM-1 (according to the DiversiLab’s patterns) and one strain co-producing NDM-1and IMP-8 to perform PCR mapping and sequencing in our study. The genetic environment surrounding NDM-1 was established by PCR mapping and subsequent sequencing of the flanking regions. Moreover, we also performed PCR mapping to elucidate the genetic structures of NDM-1 and IMP-8 in *E. cloacae* ECL-86. PCR amplicons were sequenced and the DNA sequences obtained were compared to those available in the NCBI GenBank database.

### Molecular Epidemiological Study

The clonal relationships of isolates harboring the carbapenemase-related genes were further determined by repetitive sequence-based PCR (rep-PCR) typing using the DiversiLab system. Isolates with ≥ 95% similarity were considered of the same rep-PCR type. Multilocus sequence typing (MLST) was performed using amplification of internal fragments of the seven housekeeping genes of carbapenemase-producing *E. cloacae* isolates according to the MLST^1^ website.

### Risk Factors and Clinical Outcomes of NDM-1-Producing *E. cloacae* Isolates

We conducted a retrospective case–control study to evaluate the risk factors and clinical outcomes for the isolation of NDM-1-producing *E. cloacae.* All patients with clinically obtained NDM-1-positive CR-ECL isolates were included as cases. Controls were identified as patients without NDM-1 producers and excluded if isolates harbored other carbapenemase genes, such as KPC-2, IMP-4, and IMP-8. Clinical and epidemiological data were retrieved from the medical record and clinical microbiology laboratory databases included the following parameters: demographics, underlying diseases and primary admission diagnosis, invasive devices prior to the isolation of NDM-1-producing *E. cloacae*, previous antibiotic exposure within 3 months, and clinical outcomes.

### Statistical Analysis

All analyses were performed using SPSS v.22.0 software (SPSS Inc., Chicago, IL, United States). Univariate analyses were performed separately for each of the variables. Categorical variables were calculated using a chi-square test or Fisher’s exact test as appropriate. Continuous variables were calculated using Student’s *t*-test (normally distributed variables) and Wilcoxon rank-sum test (non-normally distributed variables) as appropriate. The odds ratio (OR) and 95% confidence interval (CI) were calculated to evaluate the strength of any association. All variables with a *P*-value of ≤ 0.10 in the univariate analyses were considered for inclusion in the multivariate logistic regression model. For all calculations, statistical significance was defined at *P* < 0.05 for the two-tailed tests.

### Ethical Considerations

The data and the samples analyzed in the present study were obtained in accordance with the standards and approved by the Chongqing Medical University Institutional Review Board and Biomedical Ethics Committee. For this study, samples were collected at the Microbiology Laboratory of our hospital, with no contact with the patient. This study was retrospective and there was no patient identification performed during data collection. Therefore, the Ethics Committee determined that informed consent was not required.

## Results

### General Characteristics and Antimicrobial Susceptibility of CR-ECL Isolates

During the investigation period, a total of 138 strains were identified to be resistant to at least one of the carbapenems and met the study criteria for CR-ECL. These non-duplicated isolates were mainly cultured from urine (*n* = 55), followed by the respiratory tract (*n* = 32), wound secretion (*n* = 28), and blood (*n* = 21). As shown in **Table 1**, all CR-ECL isolates were observed in ertapenem resistance, with 60.3% of the strains still performing susceptible to meropenem and imipenem, respectively. In addition, the resistance rate to cephalosporins was relatively high in general. Of these isolates, over half of the CR-ECL strains exhibited resistance to the aminoglycoside and fluoroquinolone agents tested. However, the highest susceptibility rates to the tested antibiotics were to tigecycline (87.7%) and colistin (76.8%). Compared to carbapenemase-negative isolates, our results showed that resistance was a significantly greater proportion of *E. cloacae* isolates that were carbapenemase-positive for most tested agents, including imipenem, meropenem, cefepime, ciprofloxacin, levofloxacin, gentamycin, and tobramycin. Notably, 65.9% (91/138) of CR-ECL isolates were identified to be MDR as they were resistant to three or more classes of antimicrobial agents.

**Table 1:** Antimicrobial susceptibility of CR-ECL isolates with or without carbapenemase.

Antimicrobial agents	Total (*N* = 138)	Carbapenemase positive (*N* = 27)				Carbapenemase negative (*N* = 111)				*P*-value
	*R* (%)	*R*	MIC_50_	MIC_90_	Range	*R*	MIC_50_	MIC_90_	Range	
Ertapenem	138 (100.0)	27 (100.0)	32	128	4–128	111 (100.0)	8	32	2–64	–
Imipenem	31 (39.7)	22 (81.5)	8	32	0.25–128	9 (8.1)	0.5	2	0.25–32	**< 0.001**
Meropenem	31 (39.7)	21 (77.8)	16	128	0.5–128	10 (9.0)	0.5	2	0.25–128	**< 0.001**
Ceftriaxone	136 (98.6)	27 (100.0)	128	256	64–512	109 (98.2)	64	128	1–512	0.482
Ceftazidime	134 (97.1)	27 (100.0)	128	512	64–512	107 (96.4)	128	128	1–512	0.317
Cefepime	105 (76.1)	27 (100.0)	128	512	64–512	78 (70.3)	64	256	1–512	**0.001**
Ciprofloxacin	74 (53.6)	22 (81.5)	8	32	0.25–32	52 (46.8)	0.5	16	0.25–32	**0.001**
Levofloxacin	62 (44.9)	20 (74.1)	16	32	0.25–64	42 (37.8)	4	32	0.25–128	**0.001**
Gentamycin	71 (51.4)	24 (88.9)	32	128	1–128	47 (42.3)	1	32	1–256	**< 0.001**
Tobramycin	73 (52.9)	23 (85.2)	32	128	1–256	50 (45.0)	2	128	1–256	**< 0.001**
Colistin	32 (23.2)	7 (25.9)	1	16	1–32	25 (22.5)	1	8	1–32	0.707
Tigecycline	17 (12.3)	4 (14.8)	1	4	0.25–8	13 (11.7)	0.5	2	0.25–8	0.660

*Data are number resistant (% of resistance rates). *P*-value for comparisons of the resistance rates of carbapenemase-positive and carbapenemase-negative groups. Bold face indicates values that are significant (*P* < 0.05). R, resistance*.

### Genotypic Distribution in CR-ECL Isolates

Among the 138 CR-ECL isolates, 27 (19.6%) were detected as carbapenemase producers: 20 isolates possessed *bla*_NDM-1_, five isolates contained *bla*_IMP-8_, two isolates carried *bla*_IMP-4_, and one isolate had *bla*_KPC-2_. Notably, there was one isolate co-harboring *bla*_NDM-1_ and *bla*_IMP-8_ simultaneously. In addition to the production of carbapenemase, 76.8% (106/138) and 85.5% (118/138) of the CR-ECL isolates were positive for ESBL and AmpC genes, respectively, with 57.9% of the strains positive for both. TEM-type (57.5%, 61/106) was the most prevalent among CR-ECL isolates carrying ESBL, while EBC-type was detected among 93.2% (110/118) of AmpC-producing isolates. Additionally, fluoroquinolone and aminoglycoside genes were detected in 65.9% (91/138) and 44.9% (62/138) of all isolates, with *qnrB* (39/91) and *aac(6′)-Ib* (60/62) being the most common, respectively. All of these isolates, except the 11 CP-ECL strains, lost or had a lower expression of at least one major porin with 112 isolates for only one porin and 15 isolates for both porins. Sequencing analysis of the *ompF* and *ompC* genes of these CR-ECL isolates ruled out the occurrence of mutations or insertions. Moreover, the MICs of ertapenem were observed to have at least a twofold decrease in the presence of CCCP in 76.8% (106/138) of the CR-ECL isolates (**Table 2**).

**Table 2:** Distribution and corresponding carbapenem MIC ranges for strains with different resistance determinants.

Carbapenem resistance mechanisms	No. of isolates	MIC range (mg/L)		
		ETP	IMP	MEM
**Carbapenemase positive**				
NDM-1, loss or decreased OMPs, efflux pump	19	16–256	4–64	4–128
NDM-1+IMP-8, loss or decreased OMPs, efflux pump	1	128	128	32
IMP-8, loss or decreased OMPs, efflux pump	4	8–64	0.25–8	2–8
IMP-4, no loss or decreased OMPs, efflux pump	2	16	0.5–2	0.5–1
KPC-2, loss or decreased OMPs	1	64	8	16
**Carbapenemase negative**				
AmpC, ESBLs, loss or decreased OMPs, efflux pump	42	2–64	0.5–32	0.5–32
AmpC, ESBLs, loss or decreased OMPs	29	2–64	0.25–16	0.25–32
AmpC, loss or decreased OMPs, efflux pump	22	2–64	0.25–16	0.25–8
ESBLs, loss or decreased OMPs, efflux pump	11	2–32	0.25–2	0.25–8
ESBLs, loss or decreased OMPs	2	8	0.25–2	0.25–4
Loss or decreased OMPs, efflux pump	5	2–8	0.25–2	0.25–2

*ETP, ertapenem; IPM, imipenem; MEM, meropenem*.

### Molecular Analysis of Resistance Mechanisms

As shown in **Table 2**, all NDM-1-producing strains were resistant to three carbapenem agents, and the MIC range of these isolates was much higher than that of the isolates that carried other carbapenemases. Moreover, approximately half of the carbapenemase-positive isolates with a combination of NDM-1 and porin loss along with an active efflux pump showed the highest MICs of ertapenem, imipenem, and meropenem, while IMP-4, IMP-8, or KPC-2 producers exhibited varied carbapenem MICs. Compared to carbapenemase-positive strains, the remaining 111 isolates showed lost or decreased expression of outer membrane porins, and these isolates were often accompanied by a high prevalence of ESBL (84/111), AmpC (93/111), and efflux pump (80/111). Thus, the resistance mechanism of carbapenemase-negative isolates could be attributed to the productions of ESBL or AmpC enzymes coupled with the efflux pump and porin loss.

### Plasmid Analysis

As shown in **Table 3**, all of the transconjugants and transformants exhibited multidrug resistance phenotypes similar to those of the donor strain with reduced susceptibility to the tested carbapenems and cephalosporins, but remained susceptible to colistin and tigecycline. PCR assays confirmed that *bla*_NDM-1_ along with *bla*_CTX-M-9_, *aac(6′)-Ib-cr* and *aac(6′)-Ib* were successfully transferred by both conjugation and transformation, however, *bla*_IMP-8_, TEM, and *qnrS* were only obtained via the transformation assay. Compared to transconjugants, transformants containing two carbapenemase genes showed a twofold increase in the MIC values for ertapenem and imipenem. Although both transformants and transconjugants exhibited susceptibility to quinolones and aminoglycosides, the MICs of ciprofloxacin and levofloxacin for the transformants were four- and eightfold higher than those for the transconjugants, possibly due to the transformants containing *qnrS*. PCR-based replicon typing revealed that NDM-1 plasmids belonged to the incompatibility groups IncFIIA (*n* = 7), IncFIA (*n* = 3), IncN (*n* = 3), IncA/C (*n* = 2), and IncP (*n* = 1).

**Table 3:** Antibiotic resistance genes and susceptibility profiles in donor, *E. coli* EC600, transconjugant, *E.coli* DH5α, and transformant.

	Donor strain	Recipient strains		Transformant	Transconjugant
	EC86	DH5α	EC600		
Resistance genes	NDM-1, IMP-8, SHV, TEM, CTX-M-9, aac(6′)-Ib-cr, qnrS, aac(6′)-Ib	NA	NA	NDM-1, IMP-8, TEM, CTX-M-9, aac(6′)-Ib-cr, qnrS, aac(6′)-Ib	NDM-1, CTX-M-9 aac(6′)-Ib-cr, aac(6′)-Ib
Sequence type	ST 93	NA	NA	NA	NA
Plasmid replicon type	IncP	NA	NA	IncP	IncP
**Minimum inhibitory concentration (μg/mL)**					
Ertapenem	128	≤ 0.5	≤ 0.5	64	32
Imipenem	128	≤ 1	≤ 1	64	32
Meropenem	32	≤ 1	≤ 1	16	16
Ceftriaxone	≥ 64	≤ 1	≤ 1	≥ 64	≥ 64
Ceftazidime	≥ 64	≤ 1	≤ 1	≥ 64	≥ 64
Cefepime	≥ 64	≤ 1	≤1	16	8
Ciprofloxacin	≥ 4	≤ 0.25	≤ 0.25	1	0.25
Levofloxacin	≥ 8	≤ 0.25	0.5	2	0.25
Gentamycin	≥ 16	≤ 1	≤ 1	≤ 1	≤1
Tobramycin	≥ 16	≤ 1	≤ 1	≤ 1	≤1
Colistin	0.25	0.25	0.25	0.25	0.25
Tigecycline	≤ 1	≤1	≤ 1	≤ 1	≤ 1

### Characterization of the Genetic Environment of NDM-1 and IMP-8

For NDM-1, 15 NDM-1-producing strains could be divided into five different types (A–E) based on the analysis of genetic structures, among which type E was the most predominant (*n* = 7), followed by type D (*n* = 4), type A (*n* = 2), type B (*n* = 1), and type C (*n* = 1). Type A had a partial insertion sequence, IS5, truncating the IS*Aba125* upstream of the *bla*_NDM-1_ gene and followed by the *ble*_MBL_ (bleomycin resistance gene), the *trpF*, the *tat*, and the truncated *dct* genes. Type B harbored similar structures as type A, but it accompanied an intact IS5 element and a complete deletion of *dct* gene. Type C presented as a truncated IS*Aba125* upstream of the *bla*_NDM-1_ gene compared with Type A, but without the IS5 element. Type D carried four genetic elements downstream of the *bla*_NDM-1_ gene, including *ble*_MBL_, *trpF*, *tat*, and *dct*. Type E possessed similar genetic structures as type D, but it carried a complete deletion of *dct* gene.

**FIGURE 2:**
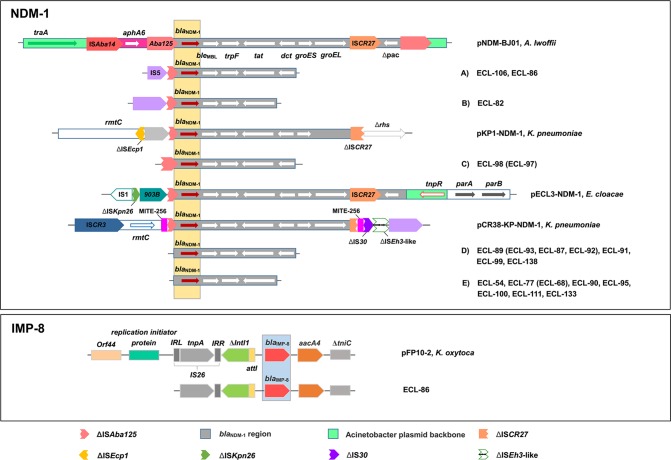
Comparison of the genetic elements surrounding the *bla*_NDM-1_ and *bla*_IMP-8_ genes identified in this study. Reference sequences: *A. lwoffii* (pNDM-BJ01, GenBank accession no. JQ001791), *K. pneumoniae* (pKP1-NDM-1, GenBank accession no. KF992018 and pCR38-KP-NDM-1, GenBank accession no. KP826710), and *E. cloacae* (pECL3-NDM-1, GenBank accession no. KC887917).

For IMP-8, the genetic structure identified from CR-ECL86 was similar to the one previously reported in *Klebsiella oxytoca* plasmids pFP10-2 (GenBank Accession No. HQ651093) from China. The *bla*_IMP-8_ gene in CR-ECL86 was preceded by a recombination site (*attI1*) and followed by an aminoglycoside acetyltransferase gene (*aacA4*) and a truncated transposase gene (Δ*tniC*). A class 1 integron (*Intl1*), located upstream of the *bla*_IMP-8_ gene in this isolate, was truncated due to the insertion of IS26. Compared to pFP10-2, the IS26 insertion in CR-ECL86 shared identical gene cassettes except for the missing left-inverted repeat sequence (LRR) in the 5′-conserved region (**Figure 2**).

### Risk Factors and Clinical Outcomes of NDM-1-Producing *E. cloacae*

To further clarify the risk factors and clinical outcomes for patients with NDM-1-producing *E. cloacae* infection, 20 NDM-1-positive *E. cloacae* cases were matched to 111 NDM-1-negative controls (**Table 4**). In the univariate analysis, patients with acquisition of NDM-1-positive *E. cloacae* were significantly more frequent in elderly, transferring from another hospital, some underlying diseases, and previous cephalosporins and carbapenem exposure (*P* < 0.05). In the multivariate analysis, transferring from another hospital (OR: 4.82, 95% CI: 1.14–20.35, *P* = 0.033) and previous carbapenem exposure (OR: 7.00, 95% CI: 1.86–26.33, *P* = 0.004) were identified as independent risk factors. For clinical outcomes, we found that the total length of hospital stay and functional status deterioration were significantly more frequent in patients with NDM-1 isolates. For in-hospital mortality, 4 (20.0%) patients infected with NDM-1 and 8 (7.2%) patients infected without NDM-1 died. However, there was no statistical significance between the cases and the controls with respect to in-hospital mortality.

**Table 4:** Univariable and multivariable analysis of risk factors and outcomes for isolation of NDM-1-positive CR-ECL isolates compared with negative controls.

Variable	NDM-1-negative *E. cloacae* (*N* = 111)	NDM-1-positive *E. cloacae* (*N* = 20)	Univariate analysis		Multivariate analysis	
			OR (95% CI)	*P*-value	OR (95% CI)	*P*-value
**Demographics, *n* (%)**		
Elderly (≥ 60 years)	21 (18.9%)	8 (40.0%)	2.86 (1.04-7.87)	**0.045**		
Male gender	52 (46.8%)	9 (45.0%)	0.93 (0.36-2.42)	1.000		
Surgical units	32 (28.8%)	9 (45.0%)	2.02 (0.76-5.34)	0.191		
Transfer from another hospital	18 (16.2%)	10 (50.0%)	5.17 (1.88-14.21)	**0.001**	4.82 (1.14-20.35)	**0.033**
Admission to ICU	22 (19.8%)	7 (35.0%)	2.18 (0.78-6.11)	0.132		
**Comorbid conditions, *n* (%)**						
Hypertension	23 (20.7%)	4 (20.0%)	0.96 (0.29-3.14)	1.000		
Malignant disease	13 (11.7%)	5 (25.0%)	2.51 (0.78-8.06)	0.152		
Cardiovascular disease	12 (10.8%)	2 (10.0%)	0.92 (0.19-4.45)	1.000		
Chronic kidney disease	16 (14.4%)	4 (20.0%)	1.48 (0.44-5.01)	0.508		
Hepatobiliary disease	38 (34.2%)	6 (30.0%)	0.82 (0.29-2.31)	0.712		
Gastrointestinal disease	25 (22.5%)	5 (25.0%)	1.15 (0.38-3.47)	0.778		
Respiratory diseases	21 (18.9%)	9 (45.0%)	3.51 (1.29-9.54)	**0.018**		
Neurological disease	16 (14.4%)	2 (10.0%)	0.66 (0.14-3.12)	1.000		
Endocrine, metabolic disease	3 (2.7%)	1 (5.0%)	1.89 (0.19-19.19)	0.489		
Vascular, hematological disease	15 (13.5%)	4 (20.0%)	1.60 (0.47-5.44)	0.491		
Hypoalbuminaemia	24 (21.6%)	9 (45.0%)	2.97 (1.10-7.98)	**0.047**		
Neutropenia	24 (21.6%)	6 (30.0%)	1.55 (0.54-4.47)	0.412		
Urinary tract infection	8 (7.2%)	1 (5.0%)	0.68 (0.08-5.74)	1.000		
Respiratory infection	27 (24.3%)	8 (40.0%)	2.07 (0.77-5.61)	0.172		
**Invasive procedures within prior 4 weeks, *n* (%)**						
Surgery in the past 6 months	29 (26.1%)	9 (45.0%)	2.31 (0.87-6.15)	0.109		
Receipt of total parenteral nutrition	8 (7.2%)	3 (15.0%)	2.27 (0.55-9.43)	0.372		
Bronchoscopes	9 (8.1%)	3 (15.0%)	2.00 (0.49-8.14)	0.325		
Mechanical ventilation	30 (27.0%)	11 (55.0%)	3.30 (1.24-8.75)	**0.013**		
Bladder irrigation	7 (6.3%)	2 (10.0%)	1.65 (0.32-8.59)	0.626		
Drainage tube	42 (37.8%)	9 (45.0%)	1.34 (0.51-3.51)	0.621		
Urinary catheter	33 (29.7%)	8 (40.0%)	1.58 (0.59-4.21)	0.433		
Tracheal cannula	25 (22.5%)	2 (10.0%)	0.38 (0.08-1.76)	0.247		
Nasal catheter	14 (12.6%)	4 (20.0%)	1.73 (0.51-5.93)	0.477		
Central venous catheter	19 (17.1%)	3 (15.0%)	0.85 (0.23-3.21)	1.000		
**Antimicrobial exposure within 3 months, *n* (%)**						
Penicillin	2 (1.8%)	0 (0.0%)	0.98 (0.96-1.01)	1.000		
Cephalosporins	37 (33.3%)	12 (60.0%)	3.00 (1.13-7.98)	**0.042**		
Carbapenem	22 (19.8%)	13 (65.0%)	7.51 (2.68-21.01)	**< 0.001**	7.00 (1.86-26.33)	**0.004**
Fluoroquinolone	17 (15.3%)	4 (20.0%)	1.38 (0.41-4.64)	0.599		
Aminoglycosides	13 (11.7%)	4 (20.0%)	1.89 (0.55-6.51)	0.294		
Glycopeptide	16 (14.4%)	5 (25.0%)	1.98 (0.63-6.20)	0.316		
Combined use of antibiotics	20 (18.0%)	4 (20.0%)	1.14 (0.34-3.77)	0.762		
**Clinical outcomes**						
In-hospital mortality, *n* (%)	8 (7.2%)	4 (20.0%)	3.22 (0.87-11.94)	0.087		
Functional status deterioration	13 (11.7%)	6 (30.0%)	3.23 (1.06-9.88)	**0.044**		
Postculture length of stay, median (IQR), days	9 (5-16)	13.5 (7.25-18.75)	NA	0.300		
Total length of hospital stay, median (IQR), days	29 (22-38)	39 (27.25-64.75)	NA	**0.015**		

*Bold face indicates values that are significant (*P* < 0.05). ICU, intensive care unit*.

### Molecular Epidemiology of CP-ECL Isolates

**FIGURE 3:**
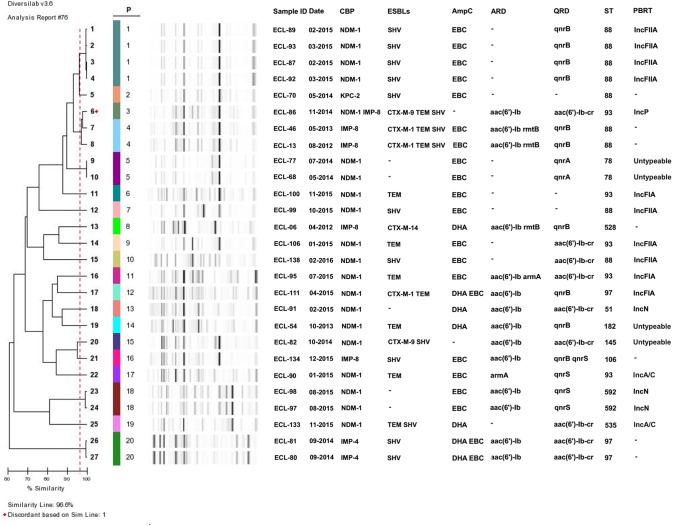
Dendrogramanalysis of DiversiLab Rep-PCR fingerprint of carbapenemase-producing *E. cloacae* isolates. A genetic similarity index scale is shown in the left of the dendrogram. Isolate number, collection data, resistance determinants, MLST, and plasmid type. CBP, carbapenemase; ESBLs, extended-spectrum β-lactamase; PBRT, PCR-based replicon typing.

The 27 carbapenemase-producing *E. cloacae* isolates belonging to 20 different clusters are shown in **Figure 3**. Of these, the main cluster (cluster 1), showing four NDM-1-carrying *E. cloacae* strains, was isolated from the respiratory department between February and March in 2015. Four smaller clusters (cluster 4, 5, 18, and 20) represented two IMP-8 isolates, two IMP-4 isolates, and four NDM-1 isolates, respectively, but these strains were collected in different wards. MLST typing revealed that ST88 was the most common among CP-ECL isolates (9/27, 33.3%), followed by ST93 (5/27, 18.5%), ST97 (3/27, 11.1%), ST78 (2/27, 7.4%), and ST592 (2/27, 7.4%).

### Description of the Outbreak Caused by NDM-1-Producing *E. cloacae* Strains

**FIGURE 4:**
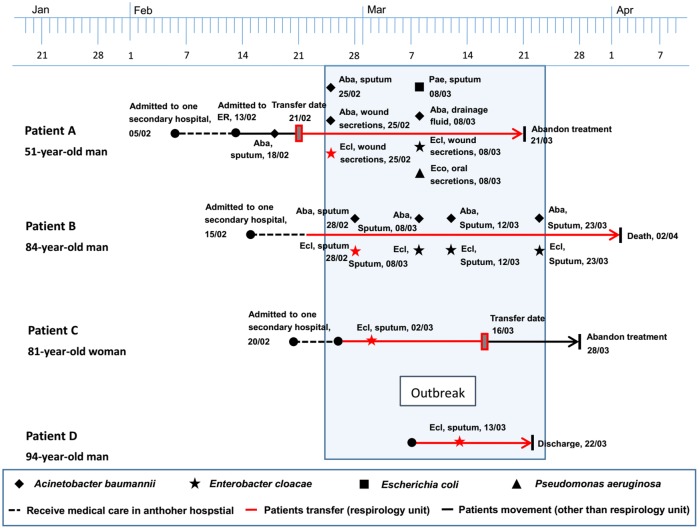
Timeline of the NDM-1-producing *E. cloacae* outbreak cases.

This outbreak involved four *E. cloacae* isolates retrieved from four hospitalized patients aged 51–94 years between February and March in 2015. Three of these four patients, who developed complications of respiratory failure and severe pneumonia, had been transferred from a secondary hospital to the respiratory unit in our setting. Strain ECL-87 was isolated from patient A on February 25, 2015, which could be the first of the outbreak NDM-1-producing isolates. Over the following 17 days, another three isolates (ECL-89, ECL-92, and ECL-93) were retrieved from three patients (patient B, C, and D) in the same department, having an average of a new NDM-1-producing *E. cloacae* infection emerging approximately every 5.6 days. In addition to the isolation of *E. cloacae*, multiple species of bacteria (including *Acinetobacter baumannii*, *E. coli*, and *Pseudomonas aeruginosa*) were recovered from the wound secretion, sputum, and drainage fluid of patients A and B. Additionally, all of the patients underwent a series of antimicrobial treatment and some invasive procedures. During the outbreak, patients A and C stopped further treatment with unimproved symptoms, and patient B died because of multiple organ dysfunction. The timeline of the outbreak investigation is illustrated in **Figure 4**.

### Nucleotide Sequence Accession Number

The sequence described in this paper has been submitted to GenBank under the Accession No. MH087224 (*E. cloacae* isolate CQECL-100).

## Discussion

In light of carbapenem resistant *E. cloacae* being widely distributed and showing the highest resistant rate among CRE in Southwest China, which was substantially different from other CRE studies in South Asia, the United States, and Europe (Nordmann et al., 2009; Pollett et al., 2014; Hsu et al., 2017), the issue of carbapenem resistant *E. cloacae* in our region deserved special concern. In this work, we have found some particularly noteworthy findings. First, our results revealed that the main mechanism of CR-ECL isolates collected in this study could be attributed to the productions of ESBL, AmpC enzymes coupled with the efflux pump and porin loss. Notably, the high prevalence (74%) of NDM-1-producing *E. cloacae* was observed among the CP-ECL isolates, with the annual increasing tendency during the study period. Previous studies have showed the emergence and spread of NDM-1-producing *E. cloacae* isolates in China, with most isolates carrying on the IncX3 and IncA/C plasmids (Yang et al., 2015). However, we reported the high prevalence of IncFIIA plasmids carrying NDM-1 (55%) in our region, revealing that this type of plasmids could be further disseminated in China.

Second, of the NDM-1 producers detected in our study, four isolates from the respiratory department showed the identical DiversiLab pattern, belonged to the same clone ST88 and displayed the same IncFIIA plasmids, providing evidence that the outbreak has emerged in our hospital. One possible source of this outbreak might be that patient A was exposed to a patient infected with NDM-1 in another hospital before the admission to our hospital. This alternative possibility may have contributed to the transmission from another unidentified colonized patient who has been discharged in the same department before the identification of these patients. To the best of our knowledge, this is the first report of a nosocomial outbreak of NDM-1-producing *E. cloacae* ST88. Most previous studies appeared to be restricted to report the sporadic cases of *E. cloacae* isolates harboring NDM-1, with diverse clones from geographic regions such as ST92 in Croatia, ST265 in Australia, and ST90, ST93, ST120, and ST177 in China (Atalić et al., 2014; Wailan et al., 2015). ST88, a globally distributed resistant clone belonging to clonal complex 23, was the most frequent ST found in *E. cloacae* isolates possessing NDM-1 in our study. The outbreak of this sequence type may provide a new model for the spread of the *bla*_NDM-1_-haboring CRE in China and should be of great concern.

Third, we reported for the first time the emergence of *E. cloacae* strain co-producing NDM-1 and IMP-8, which was isolated from a patient with urinary tract infection. This isolate showed highly resistance to many antibiotics, but remained susceptible to tigecycline and colistin. Moreover, the transmission assays of these two carbapenemase genes revealed that both NDM-1 and IMP-8 were located on separate IncP plasmids, suggesting two plasmids containing IncP replicons might be compatible in a single strain. Of greater interest was that all NDM-1 producers carried the highly conserved regions (*bla*_NDM-1_-*ble*_MBL_-*trpF*-*tat*) surrounding the *bla*_NDM-1_ gene, which were similar to that found in various NDM-1-harboring plasmids, including *K. pneumoniae* from Australia, *Acinetobacter lwoffii* from China, and *E. cloacae* from Australia (Wailan et al., 2015, 2016). This finding suggested that the transmission of NDM-1 could be widely spread among different bacterial species and geographic regions. In addition, although several reports on IMP-producing *E. cloacae* have emerged in recent years, the genetic elements with respect to *bla*_IMP-8_ in this species are still missing. Our results demonstrated that the genetic environment of this species was similar to the previous report found in *K. oxytoca* from China (Li et al., 2011), with the exception of missing a left-inverted repeat sequence in the 5′-conserved region. It should be noted that the transposon elements IS26, *intI1*, and *tniC* could facilitate the mobilization of IMP-8. Considering that the strain coproducing NDM-1 and IMP-8 may confer a higher-level resistance to multiple antibiotics that further reduce the therapeutic choices, surveillance for carbapenemase detection and infection control measures should be implemented to prevent their further spread.

Fourth, we initially performed a retrospective analysis to assess clinical predictors and outcomes for NDM-1-producing *E. cloacae.* Compared to previous studies, our results demonstrated that transferring from another hospital was an independent risk factor associated with the acquisition of NDM-1-producing *E. cloacae*. One possible explanation may be that these patients could have poor functional status and severe clinical symptoms which placed them at a greater risk for the infection caused by NDM-1-producing *E. cloacae*. Furthermore, few laboratories in Chinese secondary hospitals regularly conducted testing for CRE resistance mechanisms, especially for certain novel carbapenemases, such as NDM-1. Therefore, patients infected or colonized with NDM-1 could be the source of the horizontal transmission in different hospital environments. In addition, our study identified carbapenem exposure was a risk factor for case patients, in agreement with previous reports on the assessment of CRE (Fukuda et al., 2008; Hyle et al., 2010). Receipt of these agents could potentially produce a disruption in gastrointestinal flora and accelerate bacterial colonization, emphasizing the important role of antimicrobial stewardship in controlling NDM-1 transmission. For clinical outcomes, patients infected with NDM-1 were at a higher risk, in general, of poor outcomes than those without NDM-1. However, our results unexpectedly revealed that no statistical significance was observed in in-hospital mortality, probably due to the limited number of NDM-1-positive cases in our study.

In summary, this present study revealed the high prevalence of NDM-1 among carbapenem resistant *E. cloacae* in Southwest China. We initially reported the nosocomial outbreak caused by NDM-1-producing *E. cloacae* ST88, as well as the co-production of NDM-1 and IMP-8 in a single *E. cloacae* isolate. We also identified independent risk factors for the acquisition of NDM-1-producing *E. cloacae*, and our findings highlight an urgent need to develop effective measures to prevent and control the further spread of NDM-1 in China.

## Author Contributions

LZ, XJ, and WD designed the study. XJ, WD, WM, JY, JH, and SL performed the experiments. SY, XX, SS, JS, and CL analyzed the data. XJ wrote the manuscript.

## Conflict of Interest Statement

The authors declare that the research was conducted in the absence of any commercial or financial relationships that could be construed as a potential conflict of interest.

## Footnotes

1 http://www.mlst.net

## References

[B1] Atalić V. Z. Bedenić B. Kocsis E. Mazzariol A. Sardelić S. Barišić M. (2014). Diversity of carbapenemases in clinical isolates of *Enterobacteriaceae* in Croatia—the results of a multicentre study. *Clin. Microbiol. Infect.* 20 894–903. 10.1111/1469-0691.12635 24674100

[B2] Bustin S. A. Benes V. Garson J. A. Hellemans J. Huggett J. Kubista M. (2009). The MIQE guidelines: minimum information for publication of quantitative real-time PCR experiments. *Clin. Chem.* 55 611–622. 10.1373/clinchem.2008.112797 19246619

[B3] Carattoli A. Bertini A. Villa L. Falbo V. Hopkins K. L. Threlfall E. J. (2005). Identification of plasmids by PCR-based replicon typing. *J. Microbiol. Methods* 63 219–228. 15935499 10.1016/j.mimet.2005.03.018

[B4] Chollet R. Chevalier J. Bollet C. Pages J. M. Davin-Regli A. (2004). RamA is an alternate activator of the multidrug resistance cascade in *Enterobacter aerogenes*. *Antimicrob. Agents Chemother.* 48 2518–2523. 15215103 10.1128/AAC.48.7.2518-2523.2004 PMC434192

[B5] Clinical and Laboratory Standards Institute [CLSI] (2015). *M100-S25. Performance Standards for Antimicrobial Susceptibility Testing. 25th Informational Supplement.* Wayne, PA: Clinical and Laboratory Standards Institute.

[B6] Cornaglia G. Giamarellou H. Rossolini G. M. (2011). Metallo-beta-lactamases: a last frontier for beta-lactams? *Lancet Infect. Dis.* 11 381–393. 10.1016/S1473-3099(11)70056-1 21530894

[B7] Dai W. Sun S. Yang P. Huang S. Zhang X. Zhang L. (2013). Characterization of carbapenemases, extended spectrum beta-lactamases and molecular epidemiology of carbapenem-non-susceptible *Enterobacter cloacae* in a Chinese hospital in Chongqing. *Infect. Genet. Evol.* 14 1–7. 10.1016/j.meegid.2012.10.010 23220359

[B8] Doumith M. Ellington M. J. Livermore D. M. Woodford N. (2009). Molecular mechanisms disrupting porin expression in ertapenem-resistant *Klebsiella* and *Enterobacter* spp. clinical isolates from the UK. *J. Antimicrob. Chemother.* 63 659–667. 10.1093/jac/dkp029 19233898

[B9] Fukuda S. Kuwabara S. Yasuda M. Mizuno K. Kato T. Sugiura T. (2008). Predictors of carbapenem-resistant *Klebsiella pneumoniae* acquisition among hospitalized adults and effect of acquisition on mortality. *Antimicrob. Agents Chemother.* 52 1028–1033. 18086836 10.1128/AAC.01020-07 PMC2258527

[B10] Hsu L. Y. Apisarnthanarak A. Khan E. Suwantarat N. (2017). Carbapenem-resistant *Acinetobacter baumannii* and *Enterobacteriaceae* in South and Southeast Asia. *Clin. Microbiol. Rev.* 30 1–22. 27795305 10.1128/CMR.00042-16 PMC5217790

[B11] Hyle E. P. Ferraro M. J. Silver M. Lee H. Hooper D. C. (2010). Ertapenem-resistant *Enterobacteriaceae*: risk factors for acquisition and outcomes. *Infect. Control Hosp. Epidemiol.* 31 1242–1249. 21029005 10.1086/657138

[B12] Li B. Sun J. Y. Liu Q. Z. Han L. Z. Huang X. H. Ni Y. X. (2011). First report of Klebsiella oxytoca strain coproducing KPC-2 and IMP-8 carbapenemases. *Antimicrob. Agents Chemother.* 55 2937–2941. 10.1128/AAC.01670-10 21422214 PMC3101434

[B13] Liu C. Qin S. Xu H. Xu L. Zhao D. Liu X. (2015). New Delhi metallo-beta-lactamase 1(NDM-1), the dominant carbapenemase detected in carbapenem-resistant *Enterobacter cloacae* from Henan Province, China. *PLoS One* 10:e0135044. 10.1371/journal.pone.0135044 26263489 PMC4532496

[B14] Mahida N. Clarke M. White G. Vaughan N. Boswell T. (2017). Outbreak of *Enterobacter cloacae* with New Delhi metallo-beta-lactamase (NDM)-1: challenges in epidemiological investigation and environmental decontamination. *J. Hosp. Infect.* 97 64–65. 28552405 10.1016/j.jhin.2017.05.016

[B15] Nordmann P. Cuzon G. Naas T. (2009). The real threat of *Klebsiella pneumoniae* carbapenemase-producing bacteria. *Lancet Infect. Dis.* 9 228–236. 10.1016/S1473-3099(09)70054-4 19324295

[B16] Pollett S. Miller S. Hindler J. Uslan D. Carvalho M. Humphries R. M. (2014). Phenotypic and molecular characteristics of carbapenem-resistant *Enterobacteriaceae* in a health care system in Los Angeles, California, from 2011 to 2013. *J. Clin. Microbiol.* 52 4003–4009. 10.1128/JCM.01397-14 25210072 PMC4313239

[B17] Rasheed J. K. Kitchel B. Zhu W. Anderson K. F. Clark N. C. Ferraro M. J. (2013). New Delhi metallo-beta-lactamase-producing *Enterobacteriaceae*, United States. *Emerg. Infect. Dis.* 19 870–878. 10.3201/eid1906.121515 23731823 PMC3713825

[B18] Stoesser N. Sheppard A. E. Shakya M. Sthapit B. Thorson S. Giess A. (2015). Dynamics of MDR *Enterobacter cloacae* outbreaks in a neonatal unit in Nepal: insights using wider sampling frames and next-generation sequencing. *J. Antimicrob. Chemother.* 70 1008–1015. 10.1093/jac/dku521 25558071 PMC4356206

[B19] Tian L. Tan R. Chen Y. Sun J. Liu J. Qu H. (2016). Epidemiology of *Klebsiella pneumoniae* bloodstream infections in a teaching hospital: factors related to the carbapenem resistance and patient mortality. *Antimicrob. Resist. Infect. Control* 5:48. 10.1186/s13756-016-0145-0 27891222 PMC5114729

[B20] Wailan A. M. Paterson D. L. Kennedy K. Ingram P. R. Bursle E. Sidjabat H. E. (2015). Genomic characteristics of NDM-producing *Enterobacteriaceae* isolates in Australia and their blaNDM genetic contexts. *Antimicrob. Agents Chemother.* 60 136–141. 10.1128/AAC.01243-15 26482302 PMC4704237

[B21] Wailan A. M. Sidjabat H. E. Yam W. K. Alikhan N. F. Petty N. K. Sartor A. L. (2016). Mechanisms involved in acquisition of blaNDM genes by IncA/C2 and IncFIIY plasmids. *Antimicrob. Agents Chemother.* 60 4082–4088. 10.1128/AAC.00368-16 27114281 PMC4914633

[B22] Wu Q. Liu Q. Han L. Sun J. Ni Y. (2010). Plasmid-mediated carbapenem-hydrolyzing enzyme KPC-2 and ArmA 16S rRNA methylase conferring high-level aminoglycoside resistance in carbapenem-resistant *Enterobacter cloacae* in China. *Diagn. Microbiol. Infect. Dis.* 66 326–328. 10.1016/j.diagmicrobio.2009.10.003 19903584

[B23] Yang L. Wu A. W. Su D. H. Lin Y. P. Chen D. Q. Qiu Y. R. (2014). Resistome analysis of *Enterobacter cloacae* CY01, an extensively drug-resistant strain producing VIM-1 metallo-beta-lactamase from China. *Antimicrob. Agents Chemother.* 58 6328–6330. 25114139 10.1128/AAC.03060-14 PMC4187898

[B24] Yang Q. Fang L. Fu Y. Du X. Shen Y. Yu Y. (2015). Dissemination of NDM-1-producing *Enterobacteriaceae* mediated by the IncX3-type plasmid. *PLoS One* 10:e0129454. 10.1371/journal.pone.0129454 26047502 PMC4457825

[B25] Zhang C. Xu X. Pu S. Huang S. Sun J. Yang S. (2014). Characterization of carbapenemases, extended spectrum beta-lactamases, quinolone resistance and aminoglycoside resistance determinants in carbapenem-non-susceptible *Escherichia coli* from a teaching hospital in Chongqing, Southwest China. *Infect. Genet. Evol.* 27 271–276. 10.1016/j.meegid.2014.07.031 25107431

