# Corrigendum: Carbapenem-Resistant *E. cloacae* in Southwest China: Molecular Analysis of Resistance and Risk Factors for Infections Caused by NDM-1-Producers

**DOI:** 10.3389/fmicb.2018.02694

**Published:** 2018-11-14

**Authors:** Xiaojiong Jia, Wei Dai, Weijia Ma, Jinrong Yan, Jianchun He, Shuang Li, Congya Li, Shuangshuang Yang, Xiuyu Xu, Shan Sun, Jing Shi, Liping Zhang

**Affiliations:** Department of Laboratory Medicine, The First Affiliated Hospital of Chongqing Medical University, Chongqing, China

**Keywords:** carbapenems, resistance, *E. cloacae*, outbreak investigation, risk factor

In the original article, the legends for Figures 2, 3 were reversed. The correct legend appears below.

Figure 2. Comparison of the genetic elements surrounding the *bla*_NDM−1_ and *bla*_IMP−8_ genes identified in this study. Reference sequences: *A. lwoffii* (pNDM-BJ01, GenBank accession no. JQ001791), *K. pneumoniae* (pKP1-NDM-1, GenBank accession no. KF992018 and pCR38-KP-NDM-1, GenBank accession no. KP826710), and *E. cloacae* (pECL3-NDM-1, GenBank accession no. KC887917).

Figure 3. Dendrogram analysis of DiversiLab Rep-PCR fingerprint of carbapenemase-producing *E. cloacae* isolates. A genetic similarity index scale is shown in the left of the dendrogram. Isolate number, collection data, resistance determinants, MLST, and plasmid type. CBP, carbapenemase; ESBLs, extended-spectrum β-lactamase; PBRT, PCR-based replicon typing.

In addition, the GenBank accession number for *Klebsiella oxytoca* plasmids pFP10-2 was incorrectly written as KF732966. The correct number is HQ651093 and a correction has been made to Results, Characterization of the Genetic Environment of NDM-1 and IMP-8, Paragraph 2.

For IMP-8, the genetic structure identified from CR-ECL86 was similar to the one previously reported in *Klebsiella oxytoca* plasmids pFP10-2 (GenBank Accession No. HQ651093) from China. The *bla*_IMP−8_ gene in CR-ECL86 was preceded by a recombination site (*attI1*) and followed by an aminoglycoside acetyltransferase gene (*aacA4*) and a truncated transposase gene (Δ*tniC*). A class 1 integron *(Intl1)*, located upstream of the *bla*_*IMP*−8_ gene in this isolate, was truncated due to the insertion of IS26. Compared to pFP10-2, the IS26 insertion in CR-ECL86 shared identical gene cassettes except for the missing left-inverted repeat sequence (LRR) in the 5'-conserved region (Figure 2).

The authors apologize for this error and state that this does not change the scientific conclusions of the article in any way. The original article has been updated.

## Conflict of interest statement

The authors declare that the research was conducted in the absence of any commercial or financial relationships that could be construed as a potential conflict of interest.

